# Novel Biological-Based Strategy for Synthesis of Green Nanochitosan and Copper-Chitosan Nanocomposites: Promising Antibacterial and Hematological Agents

**DOI:** 10.3390/nano14131111

**Published:** 2024-06-28

**Authors:** Hadeer I. Mohamed, Nesrine M. R. Mahmoud, Abeer Ramadan, Abeer M. Al-Subaie, Somia B. Ahmed

**Affiliations:** 1 Department of Neuroscience Technology, College of Applied Medical Sciences in Jubial, Imam Abdulrahman Bin Faisal University, P.O. Box 4030, Jubail 35816, Saudi Arabia; hiibrahim@iau.edu.sa; 2 Department of Basic Sciences, Deanship of Preparatory Year and Supporting Studies, Imam Abdulrahman Bin Faisal University, P.O. Box 1982, Dammam 34212, Saudi Arabia; nmmahmoud@iau.edu.sa (N.M.R.M.); aramadan@iau.edu.sa (A.R.); 3 Department of Clinical Laboratory Sciences, College of Applied Medical Sciences, Imam Abdulrahman Bin Faisal University, Dammam 31441, Saudi Arabia; amnalsubaie@iau.edu.sa

**Keywords:** chitosan, nanoparticles, pomegranate peel, antimicrobial, hematological activity, copper oxide, polysaccharide

## Abstract

Two novel samples of nanoparticles based on chitosan were greenly synthesized using pomegranate peel extract. The extract served as a nanoparticle precursor, facilitating the precipitation of nanosized chitosan through the ionic gelation method. Additionally, by mixing the green chitosan nanoparticles with copper ions, a nanoscale composite of chitosan and copper oxide was also produced. Structural and morphological investigations (FTIR, XRD, SEM, EDX, and TGA analyses) were performed for greenly synthesized chitosan nanoparticles and their copper oxide composite to determine all the significant characteristics of those nanoparticles. In addition, both samples were tested using some biological investigations, such as antimicrobial activity and hematological effects. The antimicrobial tests yielded promising results for both the green chitosan nanoparticles and the CuO composite when tested using two bacterial strains and two fungal strains. Moreover, the results showed that using a similar concentration of both green-based chitosan samples resulted in a slightly larger inhibition zone and a lower minimum inhibition concentration (MIC) for the copper oxide chitosan composite compared to the chitosan nanoparticles for all microorganisms included in the test. The mean count of blood components (RBCs and platelets), clotting time, and cholesterol levels in three different blood samples were used to indicate the hematological activity of both greenly synthesized nanoparticles. The results verified a slight reduction in blood component count after the addition of green chitosan nanoparticles, but the chitosan copper oxide composite did not have a noticeable effect on the three blood samples. The chitosan nanoparticles were able to cause a considerable reduction in clotting time and cholesterol levels for all blood samples, thus acting as procoagulants. However, the mixing of CuO with chitosan nanoparticles prolonged the rate of clotting in blood samples from hypercholesteremic individuals, and thus, the mixture acted as an anticoagulant agent.

## 1. Introduction

Green approaches were utilized for the production of chitosan, a well-known cationic biopolymer, and its nanoparticles. These green approaches provide economic and environmental benefits when compared to conventional chemical and physical protocols, making them a more advantageous choice [1]. Chitosan nanoparticles are typically synthesized through chemical methods, specifically ionotrophic gelation using sodium tripolyphosphate (TPP), alongside physical methods. Although TPP is generally considered safe, it has the potential to cause variations in the size, stability, and biological properties of the resulting nanoparticles [2,3]. In sustainable processes, nontoxic, eco-friendly, and biosafe materials are employed [4]. The main goal of synthesizing green chitosan nanoparticles is to attain particle sizes smaller than 100 nm. This characteristic is vital for numerous applications that rely on the significant role of specific surface area [5]. Nanoparticles were synthesized using microorganisms such as bacteria and fungi, whose biosynthetic capabilities were harnessed during synthesis [6,7]. Moreover, the biosynthesis process involved the utilization of secondary metabolites derived from plant leaf extracts, which served as effective reducing agents [8]. Biological substances act as reducers, stabilizers, or both during the nanoparticle formation process. *Punica granatum* L., also known as “pomegranate” fruit, along with its derivatives, particularly peels, is known to contain beneficial secondary metabolites (phytochemicals). These metabolites possess various unique properties, including antimicrobial, antioxidant, anti-allergic, anti-atherosclerotic, and anti-inflammatory characteristics [9]. As a result, pomegranates and their byproducts have gained significant global recognition for their significant economic, nutritional, and medicinal potential [10]. Utilizing pomegranate peel as a capping agent contributes to reductions in cholesterol levels. This effect is attributed to the presence of its phytochemical contents, particularly polyphenols. Therefore, using pomegranate peel and its bioactive compounds could be a natural way to help lower the risk factors for heart disease [11].

Overall, nanochitosan provides significant advantages over traditional chitosan in various applications [12,13]. Its small size and increased surface area enable targeted delivery, making it effective in drug delivery systems [14]. Additionally, its biocompatibility and low toxicity make it promising for biomedical applications, including tissue engineering and regenerative medicine [15,16]. By incorporating nanomaterials such as graphene oxide, carbon nanotubes, and metal oxides like TiO_2_, ZnO [17], Fe_3_O_4_ [18], AgO [17], Au_2_O_3_ and CuO [19], the functionality of the material can be enhanced, enabling its utilization in various industries such as packaging, textiles, and environmental remediation [20,21]. The process of synthesizing chitosan–metal oxide nanocomposites involves the solution casting method for chitosan–silver oxide [17], and the chemical reduction of gold ions using X-rays as an alternative energy source in the presence of chitosan results in chitosan–Au NPs [22].

Among these notable metal oxides, considerable attention has been focused on copper oxide nanoparticles as cost-effective substitutes for noble metal nanoparticles such as silver and gold [23]. The remarkable antimicrobial [24], antiviral, and antitumor properties of copper oxide nanoparticles, coupled with their ability to enhance cell proliferation and migration and their applications in wound dressings and biocidal properties [25], have rendered them highly attractive for diverse applications [26]. The eco-friendly methods for the preparation of chitosan–copper oxide composites are important because they are less expensive than other methods [27].

It is well known that abnormal blood coagulation, injuries, surgical complications, or vascular malformations can cause severe hemorrhage, leading to shock and local tissue damage [28]. Immediate and effective hemorrhage control is crucial for saving lives. In recent times, advanced hemostatic dressings have been utilized to manage profuse bleeding [29]. Notably, chitosan-based dressings have proven effective in suppressing hemorrhage. The impact of chitosan on various blood components, including red blood cells (RBCs), coagulation factors, and platelets, must be considered. Surprisingly, the hemocompatibility compatibility of chitosan remains under investigation. When in contact with whole blood, chitosan forms a coagulum, leading to distinct platelet adhesion on its surface within a short timeframe. Notably, clotting time is reduced by 40% compared to whole blood alone. Consequently, chitosan has a practical application as a hemostatic dressing in clinical settings to promote wound healing rather than as a blood-contact medical device [30].

This research article focuses on two main objectives. Firstly, it describes the synthesis of green nanochitosan, a versatile biopolymer, using a green extract from pomegranate peel. The goal is to develop an environmentally sustainable approach to nanochitosan synthesis by eliminating the use of chemicals or traditional agents like tripolyphosphate (TPP). Secondly, this study aims to outline the creation of a novel bifunctional composite material using a combination of the unique properties of biogenic copper oxide nanoparticles and the versatility and biocompatibility of nanochitosan. This article also details an investigation of the antimicrobial properties of these nanoparticles and an evaluation of their potential for promoting healing processes.

## 2. Experimental Work

### 2.1. Plant Extract Preparation (Pomegranate Peel Extract)

The natural extract was prepared by combining an equivalent of 40 g of raw pomegranate peel powder with one liter of distilled water. The mixture was subjected to boiling for a duration of 15 min, followed by cooling and subsequent filtration to obtain the desired natural extract.

### 2.2. Synthesis of Green Chitosan Nanoparticles (PP–Cs–NPs)

To prepare a sample of chitosan nanoparticles (PP–Cs–NPs), the process involved dissolving half a gram of commercial chitosan in one percent glacial acetic acid until complete dissolution. Fifty milliliters of pomegranate peel extract were carefully added to the chitosan solution while it was being heated at 80 °C, and the mixture was continuously stirred for an hour. Subsequently, the mixture was washed multiple times with distilled water and filtered using decantation. Finally, the sample was left to dry overnight in an oven at 80 °C.

### 2.3. Synthesis of Green Chitosan–Copper Oxide Composite (PP–CS–CuO–NPs)

The synthesis of the second sample (PP–Cs–CuO–NPs) involved a three-step process. Firstly, half a gram of commercial chitosan was fully dissolved in a one percent glacial acetic acid solution. Secondly, 50 millimoles of anhydrous copper sulfate was added to the previous mixture, followed by the careful addition of 50 mL of pomegranate peel extract. This step was conducted at a temperature of 80 °C, with continuous stirring for an hour. Finally, the mixture was washed multiple times with distilled water, filtered using decantation, and dried overnight in an oven at 80 °C.

### 2.4. Characterization

#### 2.4.1. Structural Assessment

To examine the characteristic functional groups of the synthesized nanoparticles, FTIR spectra using Shimadzu Fourier Transform Infrared spectrophotometer were used [31]. The thermal stability of all greenly synthesized nanoparticles was evaluated through thermo-gravimetric analysis (TGA-DTA7300, Exstar, Hitachi, Tokyo, Japan) at ambient temperature. An X-ray powder diffractometer using (Shimadzu, Kyoto, Japan) XRD with Cu Kα radiation (λ = 1.5418 Å) at a scanning speed of 0.2 s was used to identify the characteristic peaks of both synthesized nanoparticles (details are provided in the Supplementary Materials).

#### 2.4.2. Morphological Characterizations

SEM (FEI, Inspect S 50, Czech Republic) was used to examine the morphology and arrangement of the synthesized nanoparticles. Furthermore, using energy dispersive X-ray spectroscopy, the EDAX analyses were conducted with an EDX-8000 instrument (Shimadzu, Kyoto, Japan) for elemental analysis and identification of the synthesized nanoparticles [32] (details are provided in the Supplementary Materials).

### 2.5. Antibacterial Test

The susceptibility tests were performed according to the guidelines provided by the National Committee for Clinical Laboratory Standards (NCCLS, 1993). Screening tests to determine inhibition zones were performed using the well diffusion method [33] (details are provided in the Supplementary Materials).

### 2.6. Hematological Test

#### 2.6.1. Chitosan Solution Preparation

The chitosan solution was prepared by dissolving 10 mg/mL of each of the 2 samples, the PP–Cs–NPs and PP–Cs–CuO–NPs, in 1% acetic acid, with continuous stirring at ambient temperature for 2 h.

#### 2.6.2. Blood Collection

The blood samples were obtained from individuals with overall good health, diabetes, or hypercholesterolemia.

The Institutional Review Board of Imam Abdulrahman Bin Faisal, Kingdom of Saudi Arabia, granted ethical approval for this research (reference number: IRB-2020-03-339). This study adhered to the principles outlined in the Declaration of Protecting Human Research Participants Online Training (PHRP No. 2852904) for conducting research involving human subjects. Additionally, all participants provided informed consent by signing a consent form prior to their involvement in the study (details are provided in the Supplementary Materials).

#### 2.6.3. Complete Blood Count (CBC) and Coagulation Time (BCT)

Hematological analysis, including erythrocyte count (RBC, count/mm^3^) and platelet count (PLT, count/mm^3^), was conducted using the CELLTAC hematology analyzer. The complete blood count and coagulation time were assessed by introducing half a mL of the PP–Cs–NPs and PP–Cs–CuO–NPs solutions to one and a half ml of each blood sample, each of which had been previously divided into tubes containing 3.8% sodium citrate. Following a 5-min incubation period in a water bath at 37 °C, the blood samples were analyzed using the CELLTAC instrument. To observe blood coagulation, the tube was gently inclined at 30-s intervals until the blood clot formed. The coagulation time was recorded when no blood flow was observed upon perpendicularly tilting the tube. CBC was analyzed using the same procedure. The mixture was then incubated in a water bath at 37 °C for 5 min.

## 3. Results and Discussion

### 3.1. Characterization of Chitosan Nanoparticles

#### 3.1.1. FTIR Analysis

**Figure 1:**
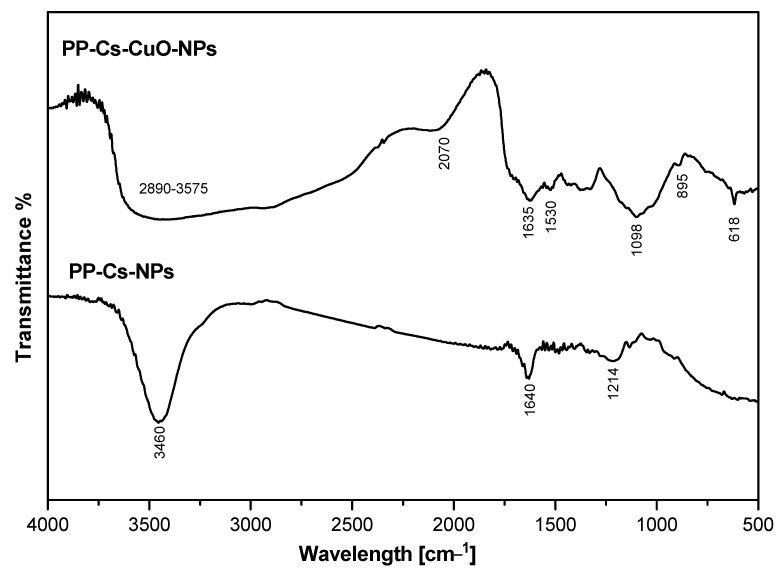
FTIR for green chitosan PP–Cs–NPs and green chitosan–CuO nanocomposites (PP–Cs–CuO–NPs).

Figure 1 presents the spectra of both PP–Cs–NPs and PP–Cs–CuO–NPs, which were obtained using FTIR. In the spectrum of PP–Cs–NPs, a prominent peak at 3400 cm^−1^ is observed, indicating the overlap between the N–H of chitosan, the O–H stretching of phenolic compounds, and the polysaccharides present in the pomegranate peel extract. Another peak appeared at 1640 cm^−1^, indicating the existence of a –CONH group characteristic of chitosan. Additionally, a sharp peak observed at 1214 cm^−1^ corresponds to a C–O group characteristic of phenolic compounds found in the pomegranate peel extract [34,35]. On the other hand, the spectrum of the PP–Cs–CuO–NPs exhibited several bands in different regions. A broad band ranging from 2890 to 3575 cm^−1^ could be attributed to the merging of bands of the N–H group of chitosan, the O–H stretching of the organic compounds from the pomegranate peel, which is found in the range of 3400 cm^−1^, and a band of C–H asymmetric stretching at 2920 cm^−1^ [36]. The vibrational bending of an N–H group was observed at 1530 cm^−1^. Furthermore, a band at 2070 cm^−1^ indicates the existence of silicon compounds. Two additional bands in the range between 1635 and 1098 cm^−1^ represent the existence of the –CONH group of chitosan and the C–O group of phenolic compounds and polysaccharides in pomegranate peel, respectively. A small peak corresponding to the stretching vibration of metal oxide was found at 895 cm^−1^ [37]. Evidence of CuO–NPs formation was found in the sharp band at 618 cm^−1^ [19,38,39]. Finally, the difference between the spectra of CuO–NPs and PP–Cs–CuO–NPs was mainly indicated by the existence of bands at 895 and 618 cm^−1^ that are characteristic of the metal oxide group. Also, the peaks of PP–Cs–CuO–NPs at 2920, 2070, and 1530 cm^−1^ provide evidence of more organic matter from the pomegranate peel extract, which may be needed to precipitate CuO–NPs. Based on this data, it can be inferred that pomegranate polysaccharides serve as a highly effective alternative to conventional TPP as a nanoparticle precursor [5].

#### 3.1.2. XRD Analysis

**Figure 2:**
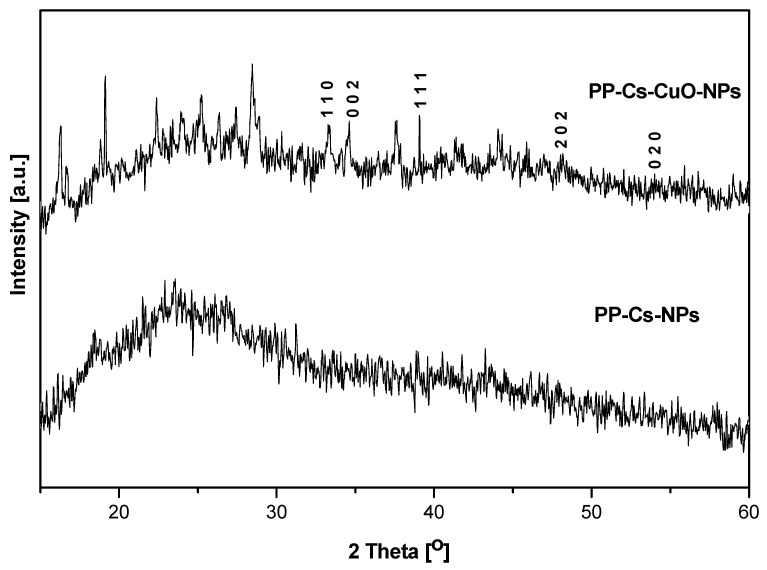
XRD patterns for green chitosan (PP–Cs–NPs) and green chitosan–CuO nanocomposites (PP–Cs–CuO–NPs).

Figure 2 exhibited no distinct peaks in the XRD diffractogram of PP–Cs–NPs, indicating the amorphous nature of synthesized nanoparticles. This characteristic enhances the sorption properties of the material [3]. However, PP–Cs–CuO–NPs showed weak CuO pattern peaks of 2θ = 32.9°, 35.0°, 38.5°, 48.0°, and 53.2°, corresponding to (−110), (002), (111), (−202), and (020) reflections, respectively [40]. These peaks confirmed the presence of the CuO phase (monoclinic structure, JCPDS card no. 45-0937). Some peaks representing impurities from pomegranate peel capping were overlaid on the broad, amorphous band of the chitosan matrix [41]. These peaks suggest the existence of K_2_O and K_2_CO_3_ at 2θ = 25.2°, 28.2°, and 40.7° (JCPDS 77-2176 and 87-0730), while the weak peaks at 2θ = 25.0°, 32.9°, 42.1°, and 51.26° were attributed to SiO_2_, Fe_2_O_3_, P_2_O_5_, and carbon (JCPDS 41-1413, 33-0664, 5-0488, 75-1621 and 34-0941) [19].

#### 3.1.3. TGA and DTA

**Figure 3:**
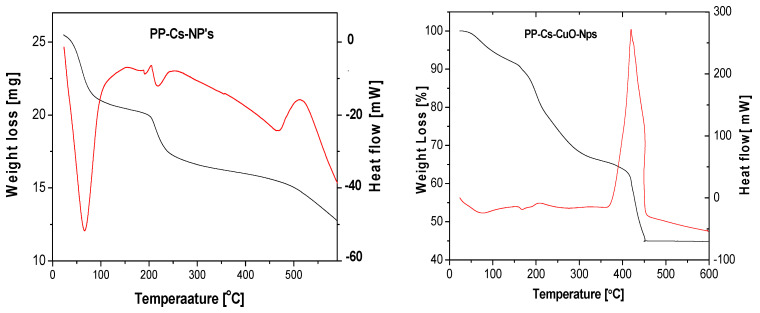
TGA (black) and DTA (red) for green chitosan (PP–Cs–NPs) and green chitosan–CuO nanocomposites (PP–Cs–CuO–NPs).

To explore the thermoanalytical characteristics of the green chitosan samples, thermal analysis was performed across a controlled temperature range. The thermal gravimetric analysis (TGA) and differential thermal gravimetric (DTA) curves of PP–Cs–NPs and PP–Cs–CuO–NPs are depicted in (Figure 3). Table 1 provides the temperature values corresponding to each stage of thermal decomposition, along with their respective weight losses. The poor thermal stability of conventional chitosan represents a significant challenge, especially for its biomedical applications [42,43]. However, both samples examined in this study (PP–Cs–NPs and PP–Cs–CuO–NPs) exhibited improved thermal stability compared to regular chitosan.

During the initial stages of TGA, the decrease in mass observed with chitosan nanoparticles can be attributed to the drying process, where residual water bound to the polar groups in chitosan nanoparticles is lost. This weight loss is not associated with any chemical reactions and is primarily due to the removal of water molecules. It is evident that PP–Cs–NPs undergo a first weight loss of approximately 16.7% between 25 °C and 87 °C, primarily due to dehydration.

The high weight loss during the dehydration stages may be due to the increased hydrophilicity of PP–Cs–NPs compared to regular chitosan. The subsequent decomposition of PP–Cs–NPs takes place in the second and third stages in which a residue of about 42.8% at 600 °C is left behind. On the other hand, PP–Cs–CuO–NPs exhibited a different thermal behavior, with water elimination occurring within a temperature range from 25 °C to 117 °C with 8.9% weight loss. The decomposition of chitosan and the capping green extract residue appeared in the following two stages in a temperature range of 117–450 °C. Figure 3 confirms the improved stability of PP–Cs–CuO–NPs by showing no significant mass loss beyond 450 °C. The material exhibits a residue of 45%, as shown in Table 1. This suggests that PP–Cs–CuO–NPs maintain their structural integrity and do not undergo further decomposition at higher temperatures. This finding suggests that these modified forms of chitosan show potential in overcoming the thermal stability challenges encountered in biomedical applications.

**Table 1:** Sample weight loss and thermal degradation stages.

Sample	Degradation Stage	Temperature Range (°C)	Peak Temperature (°C)	% Weight Loss
PP–Cs–NPs	First	25–87	68	16.7%
	Second	87–240	218	31.5%
	Third	240–600	470	51.8%
PP–Cs–CuO–NPs	First	25–117	79	8.9%
	Second	117–314	200	23.1%
	Third	314–451	415	54.8%

#### 3.1.4. SEM and EDX

**Figure 4:**
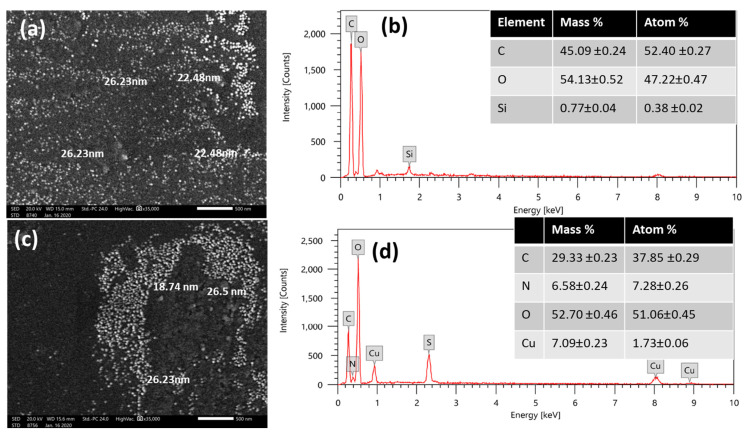
SEM images of (**a**) green chitosan (PP–CS–NPs), (**c**) green chitosan–CuO nanocomposites (PP–CS–CuO–NPs); EDX elemental % (**b**) green chitosan (PP–CS–NPs), and (**d**) green chitosan–CuO nanocomposites (PP–CS–CuO–NPs).

Figure 4 reveals the morphological distribution (size, shape) and the elemental structure of synthesized samples. Figure 4a shows a uniform dispersion of exemplary spherical nanoparticles that exhibit a narrow particle size distribution in an average range from 22.5 nm  ±  1.2 nm to 26.2 nm  ±  1.3 nm for the sample of PP–Cs–NPs and from 18.7 nm  ± 1.0 nm to 26.5 nm  ±  1.3 nm for the sample of PP–Cs–CuO–NPs. Meanwhile, the SEM image (Figure 4c) depicts two distinct types of nanoparticle aggregates on the surface within the same particle size range. Furthermore, Figure 4b,d verify that PP–Cs–NPs primarily consisted of carbon and oxygen, whereas PP–Cs–CuO–NPs comprised carbon, oxygen, nitrogen, and copper.

### 3.2. Evaluation of Antimicrobial Activity of Green PP–Cs–NPs and PP–Cs–CuO–NPs

**Figure 5:**
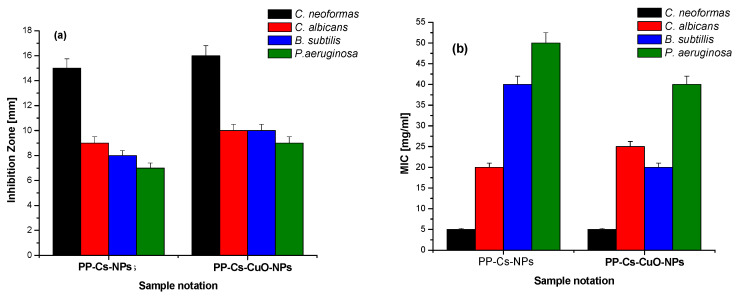
(**a**) Inhibition zone [mm] and (**b**) minimum inhibitory concentrations [mg/mL] of green chitosan (PP–Cs–NPs) and green chitosan–CuO nanocomposites (PP–Cs–CuO–NPs).

The antimicrobial activity of both green PP–Cs–NPs and PP–Cs–CuO–NPs was evaluated using the gram-positive bacterium *Bacillus subtilis* (*B. subtilis*) and the gram-negative bacterium *Pseudomonas aeruginosa* (*P. aeruginosa*), as well as two types of fungi, *Cryptococcus neoformans* (*C. neoformans*) and *Candida albicans* (*C. albicans*). The diameter of the inhibition zone and minimum inhibitory concentration (MIC) were measured for each sample. Interestingly, both samples demonstrated good activity against the microorganisms tested (Figure 5). The results demonstrate that the antimicrobial activity of PP–Cs–CuO–NPs resulted in slightly larger inhibition zones (16, 10, 10, and 9 mm) compared to the antimicrobial activity of PP–Cs–NPs (15, 9, 8, 7 mm) at the same concentration for all microorganisms used, indicating enhanced antimicrobial activity, as previously found in the literature [44].

Also, the MIC values showed that both chitosan samples exhibited antimicrobial activity. PP–Cs–CuO–NPs generally demonstrated MIC values that were comparable to or slightly lower than PP–Cs–NPs, suggesting similar or improved antimicrobial efficacy. It is important to note that the MIC results generally align with results of the diameter of the inhibition zone, indicating a correlation between the two measures of antimicrobial activity. However, it is noteworthy that for the *C. albicans* fungus, PP–Cs–NPs exhibited a lower MIC value (20 mg/mL) compared to that of PP–Cs–CuO–NPs (25 mg/mL). This discrepancy suggests that while PP–Cs–NPs might have a smaller inhibition zone diameter (9 mm) against *C. albicans*, a lower concentration is needed to effectively inhibit fungal growth. This discrepancy could potentially be attributed to variations in the susceptibility of *C. albicans* to different chitosan formulations [27].

The findings revealed a marginally reduced antimicrobial efficacy for the green samples compared to the previously prepared TPP-based samples, with the exception of *C. neoformans* treated with PP–Cs–NPs, which exhibited superiority (15 mm) over the chemical-based chitosan nanoparticles from previous work (9 mm) [19].

Our study introduces a groundbreaking green synthesis method, utilizing raw pomegranate peel extract instead of TPP, thereby eliminating the requirement for chemicals and complex extraction processes. This substitution marks a significant advancement, as TPP has traditionally been a key precursor in chitosan synthesis. Additionally, despite a minor decline in antimicrobial activity, our chitosan samples, which were synthesized in an environmentally friendly manner, demonstrated comparable antimicrobial effectiveness, affirming the practicality and efficacy of employing nanochitosan and its composites derived from natural extracts for antimicrobial applications.

### 3.3. Hematological Analysis

**Figure 6:**
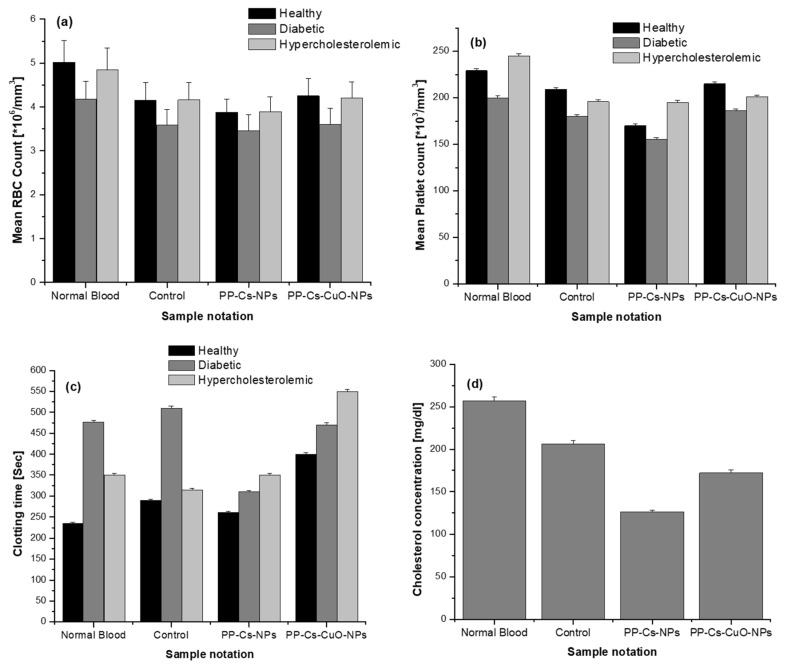
The effect of green chitosan (PP–Cs–NPs) and green chitosan–CuO nanocomposites (PP–Cs–CuO–NPs) on (**a**) mean RBC count, (**b**) mean platelet count, (**c**) clotting time, and (**d**) cholesterol concentration in blood samples from healthy, diabetic, and hypercholesteremic individuals.

Compared to control blood samples, slight reductions in the mean blood component counts were observed in all blood samples after the addition of PP–Cs–NPs; by contrast, the addition of PP–Cs–CuO–NPs did not result in noticeable changes in the RBC or platelet counts in hematological specimens from healthy, diabetic, and hypercholesteremic individuals., as shown in Figure 6a,b. The addition of PP–Cs–NPs led to a 6.5% reduction in the mean RBC count in blood specimens from healthy and hypercholesterolemic individuals compared to a 4% reduction in blood samples from diabetic individuals (Figure 6a). Decreases in platelet count of 18.7% and 14% were observed in samples from healthy and diabetic individuals, respectively, without any noticeable change in platelet count in blood samples from hypercholesterolemic individuals. 

Figure 6c demonstrates the in vitro effects of both biosynthesized samples on the coagulation time of all tested blood samples. PP–Cs–NPs reduced the time of blood clotting for healthy and diabetic blood specimens, whereas the clotting time of blood samples from hypercholesteremic individuals was increased. PP–Cs–CuO–NPs increased the clotting time of blood samples from healthy and hypercholesterolemic individuals. That means that both synthesized nanoparticles may be able to accelerate the wound healing process in diabetic individuals by reducing clotting time. Figure 6c,d show the effect of both synthesized samples, especially PP–Cs–NPs, on cholesterol concentration reduction in blood samples from hypercholesteremic individuals.

Chitosan has a crucial impact on the prevention of atherosclerosis by transporting glucosamine groups to the cell via glucose receptors. Previous studies have verified that the surface of the human blood cell contains approximately 107 negative ionic groups due to the presence of a carboxylic acid group of N-acetylneuraminic acid at the peripheral end of a protein called glycophorin that penetrates the lipid bilayer, which is responsible for the majority of the negative charges [45]. The net positive charge of chitosan nanoparticles (Cs–NPs), which relies on the degree of deacetylation (DD) and the number of protonated amine groups, is responsible for their hemostatic properties [46,47]. These amine groups promote the interaction between the negatively charged blood components (red blood cells and platelets) and positively charged chitosan (13 mV), enabling chitosan to form a spatial structure resembling a mesh, which facilitates blood clot formation (Figure 6a,b). Previous studies have demonstrated that chitosan with moderate DD exhibits the most significant procoagulant effect [48].

On the other side, copper nanoparticles play a crucial role in inhibiting and masking the inflammatory effect of chitosan, as well as assisting copper-dependent enzymes in collagen synthesis, which aids in wound healing [49]. The wound healing property of nano-copper (nCu) is enhanced when combined with chitosan. Chitosan is known to be polycationic in acidic media, allowing it to bind easily with metallic ions such as Cu [20].

Additionally, a *P. granatum* extract led to prolonged clotting time without any noticeable change in the blood component count, which could be attributable to the existence of anthocyanidins in *P. granatum* that play a vital role in suppressing cyclooxygenase [50] or diminishing the level of fibrinogen [51], thus preventing platelet aggregation.

The nonsignificant reduction in the RBC and platelet counts after the addition of PP–Cs–CuO–NPs (Figure 6a,b) is attributed to the chelating effect of Cu, which suppresses the interaction of chitosan nanoparticles and blood components. This verifies that the incorporation of CuO with peel extract in Cs–NPs serves as an anticoagulant that inhibits thrombin and intrinsic coagulation factor [52]. The significant reduction in cholesterol levels achieved by adding PP–Cs–NPs may be attributed to the potential interaction between the positive nanochitosan particles and the lone pair electrons of the hydroxyl group in cholesterol [53]. The preparation of PP–Cs–NPs and PP–Cs–CuO–NPs through the gelation of pomegranate peel extract in an acidic environment results in the formation of a cationic form of chitosan (–NH_3_^+^), as indicated by the zeta results (details are provided in the Supplementary Materials) [54]. The cationic chitosan binds to the hydroxyl group of cholesterol, creating a composite that effectively conceals the cholesterol molecules during the testing process. This composite is believed to inhibit the absorption of cholesterol in vivo.

## 4. Conclusions

This study investigated the successful synthesis of two types of nanoparticles, PP–Cs–NPs and PP–Cs–CuO–NPs, using a fully green technique. It introduced a novel approach to replacing TPP as a nanoparticle precursor by utilizing polysaccharides from pomegranate peel extract, known for its sustainability, eco-friendly nature, and green attributes. The characterization of both PP–Cs–NPs and PP–Cs–CuO–NPs confirmed their spherical uniformity, positive charge, and particle size ranging from 20 to 27 nm.

Biological evaluations of these newly synthesized nanoparticles (PP–Cs–NPs and PP–Cs–CuO–NPs) demonstrated their efficacy as bifunctional agents (antimicrobial agents and wound healers). Both PP–Cs–NPs and PP–Cs–CuO–NPs exhibited significant inhibition of bacterial and fungal growth, with the highest inhibition observed against the fungus *C. neoformans* at MIC values of 5 mg/mL. Furthermore, PP–Cs–CuO–NPs displayed a slightly higher antimicrobial activity compared to PP–Cs–NPs. Additionally, both nanoparticles reduced clotting time in blood samples from diabetic patients.

Finally, the study revealed a crucial role of PP–Cs–CuO–NPs and PP–Cs–NPs in reducing cholesterol levels in blood samples from both normal and hypercholesterolemic individuals through the creation of a hybrid structure between nanochitosan and cholesterol molecules.

## Data Availability

The original data presented in the study are openly available in the article and Supplementary Materials.

## Supplementary Materials

The following supporting information can be downloaded at: https://www.mdpi.com/article/10.3390/nano14131111/s1, Supplementary Files S1 and S2.
