# Supplementary material for: Novel Biological-Based Strategy for Synthesis of Green Nanochitosan and Copper-Chitosan Nanocomposites: Promising Antibacterial and Hematological Agents
Source: Nanomaterials (Basel). 2024 Jun 28;14(13):1111. doi: 10.3390/nano14131111 (PMC11243605; doi:10.3390/nano14131111)

# Supplementary File S1

## S1.1. Structural Assessment

Shimadzu Fourier Transform Infrared spectrophotometer was used to record the spectra at 4.0 $cm^{-1}$ resolution. To enhance the signal-to-noise ratio, 64 scans were accumulated. For the analysis the dried powdered sample was compressed with KBr and tested simultaneously . The thermal stabilty of all greenly synthesized nanoparticles were evaluated through thermo-gravimetric analysis (TGA -DTA7300, Exstar) at ambient temperature. The analysis was conducted in atmospheric air with a uniform heating rate 10 °C/min within the range 25–600 °C, which were placed in an alumina crucible.

## S1.2. Morphological characterizations

SEM (FEI, ISPECT S50, Czech Republic) was used to examine the morphology and arrangement of the synthesized nano-particles. It was operated at 20 kV with approximately 10 mm working distance . The samples were mounted on a metallic stub using double-sided adhesive tape. Images were captured at various different magnifications to allow for detailed visual inspection and to note important features of the specimens. Furthermore, energy dispersive X-ray spectroscopy the EDAX analyses were conducted using an EDX-8000 instrument (Shimadzu) for elemental analysis and identification of the synthesized nanoparticles.

## S1.3. Anti-Bacterial test

The Susceptibility tests were pursuing the guidelines provided by the National Committee for clinical laboratory Standards (NCCLS, 1993). Screening tests to determine inhibition zones were performed out by the well diffusion method[18]. The inoculum suspension was prepared by harvesting colonies grown overnight on an agar plates, which were then inoculated into Mueller-Hinton broth (or malt broth for fungi). A sterile swab was immersed in the suspension and used to inoculate Mueller-Hinton agar plates (or malt sugar plates for fungi). The samples being tested were dissolved in dimethyl sulfoxide (DMSO) at different concentrations (10, 5, 2.5 mg/ml). After incubation for 24 hours at 37 ºC, the inhibition zones were measured around each well. Adequate controls using DMSO were included in the experiment.

## S1.4. Hematological test

**S1.5. Blood Collection**

10ml blood sample was collected using 21-gauge needles with three-way stop-cocks to minimize tourniquet pressure from the antecubital vein. For all the studies except for blood coagulation time, the collected blood was divided into three tubes, containing anticoagulant 3.8% sodium citrate. The selection of subjects for each blood type was based on specific criteria. The healthy blood samples were chosen based on normal platelet counts, while the diabetic blood samples were selected with high blood glucose levels ranging from 180-200 mg/dl. Similarly, the hypercholesterolemia blood samples were chosen with LDL cholesterol levels ranging from 160-180 mg/dl.

Supplementary File S2

Zeta values

| **Sample** | **Zeta- potential** |
|---|---|
| PP-Cs-NPs | 13.1 mV ± 6.3 mV |
| PP-Cs-CuO-NPs | 7.6 mV ± 5.8 mV |

Histogram of SEM

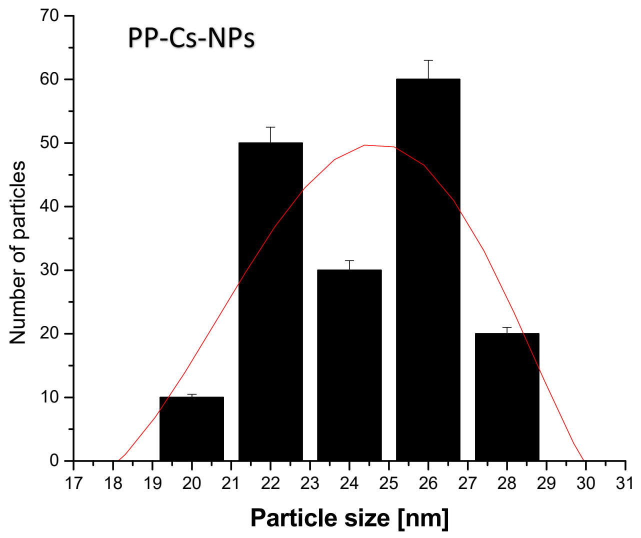


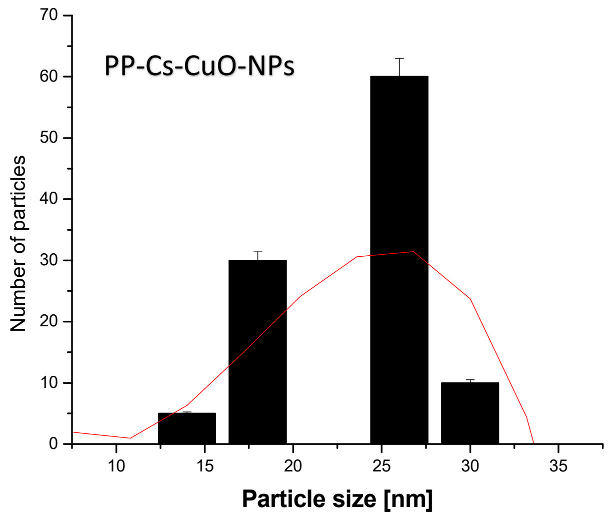

Supplement: Supplementary file 1 [file nanomaterials-14-01111-s001.zip › nanomaterials-3054255-supplementary.pdf]
